# Biocomposite Films Reinforced With Nano Navy Bean Starch: Application and Characterization

**DOI:** 10.1002/fsn3.72067

**Published:** 2026-07-07

**Authors:** Nora Ali Hassan, Aijun Hu, Ammar Badran Altemimi, Rawaa Houri Tlay, Qian Zhang, Bing Lu, Shiwei Liu, Tarek Gamal Abedelmaksoud

**Affiliations:** ^1^ College of Food Science and Engineering Tianjin University of Science & Technology Tianjin China; ^2^ Food Science Department, Faculty of Agriculture Cairo University Giza Egypt; ^3^ Department of Food Science, College of Agriculture University of Basrah Basrah Iraq; ^4^ Department of Food Science, College of Agricultural Engineering Damascus University Damascus, Damaskus Syria

**Keywords:** biocomposite films, biodegradability, dynamic light scattering, optical properties, TGA, WVTR

## Abstract

In this study, enzymatically ultrasonicated starch biocomposite film (E‐USCF) was produced by reinforcing enzymatically ultrasonicated starch nanoparticles (E‐US) with native navy bean starch (NS) in different concentrations (0.5%, 1%, 2%, 5%, and 10%). After that, they were characterized in terms of physicochemical, mechanical, and thermal properties and compared to native starch biocomposite films (NSCF). The E‐USCF samples showed high solubility, pH, breaking strength, and thickness by increasing the concentration of E‐US samples while reducing the water vapor transmission rate (WVTR), moisture content, swelling characteristics, and transparency. Moreover, the biodegradability rate reached 87% within 8 days by elevation of E‐US up to 10%. As a consequence, thermogravimetric analyses (TGA) confirmed the same results, as E‐USCF 10% had an ash residual of 19.88% compared to the NSCF sample at 23.95%. Furthermore, lowering tensile stress and strain of biocomposite film analyses were confirmed by the elevation of incorporation of E‐US up to 10%. In summary, E‐US particles acted as a structural reinforcing agent and improved E‐USCF's properties.

## Graphical abstract

This study developed enzymatically ultrasonicated starch composite films (E‐USCF) reinforced with nano starch particles. The films exhibited higher solubility, pH, strength, thickness, and biodegradability, with reduced moisture, WVTR, swelling, and transparency. TGA supported improved thermal behavior. The 10% E‐USCF showed optimal performance, confirming potential as biodegradable packaging material.

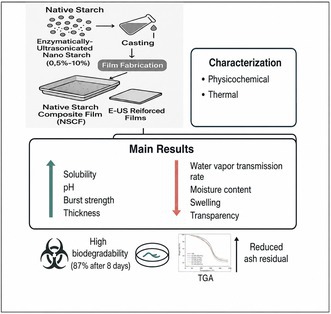

## 1 Introduction

Starch biocomposite films have been used widely for both food and non‐food purposes. According to Singh et al. (2022), starches derived from a variety of agricultural resources, including rice, sorghum, beans, potatoes, and corn, have shown promise as environmentally friendly food packaging materials. In contrast to legume starches, little is known about their production, consumption, and physiochemical and structural characteristics (Keskin et al. 2022). According to (Hassan et al. 2025; Do et al. 2023), Because of the strong correlation between their cotyledon cell structures and starch digestion in vitro, navy beans had two to three times more protein than any grain and intact tissue or cellular structures encasing starch granules. As reported by Marquezi et al. (2016), bean starches contained high amylose concentrations 45.32%–51.11%. But as described by Zhang and Li (2021), navy bean starches had hydrophilic nature changed the properties of the starch‐based biocomposite films. According to the study conducted by Roy et al. (2020), this greatly limited their usefulness as food packaging materials because of how much moisture they absorbed from the environment causing poor mechanical efficiency such as breaking strength and elevation in its biodegradability.

According to Folino et al. (2023), biodegradability of biocomposite films was defined as the degradation of a film structure with organic wastes in the presence of microorganisms. Thus, bacteria utilized sugar from starch as a source of energy, resulting in the breakage of starch polymers. Thereby, utilization of nanotechnology in production of food biocomposite films was considered a viable substitute for conventional packaging by greatly enhancing mechanical, thermal and barrier qualities (Honarvar et al. 2016; Marín‐Silva et al. 2019). For example, advances in biocomposite films through chemical modifications (such as cross‐linking and phosphorylation) were improving mechanical strength and moisture resistance of biocomposite films. Furthermore, as described by Wang et al. (2020), the excessive amounts of nano starch contained high amylose amount were touched by hydrogen bonding as their significant amounts of hydroxyl functional groups through cross‐linked agents such as plasticizers could improve film flexibility by decreasing intermolecular pressures and improving the mobility of polymeric chains. Nevertheless, a reduction in stiffness, tensile strength, and gas barrier qualities frequently coexisted with this plasticizing action (Akachat et al. 2025).

Hence, according to the study of Hassan et al. (2025), who modified navy bean starch via enzymatic debranching and ultrasonication processes. So, in this study, it was used in the production of food packaging biocomposite films in different concentrations (0%, 0.5%, 1%, 2%, 5%, and 10%). Nevertheless, the films were characterized in terms of mechanical properties, weight loss, morphology, physicochemical characteristics, and thermal behavior, offering a comprehensive understanding of their potential for use in sustainable packaging solutions.

## 2 Materials and Methods

### 2.1 Materials

In this study, according to Hassan et al. (2025), the starch was extracted from navy beans that were purchased from Kunming Shimingxuan E‐commerce Co. Ltd. (China). In addition, in this study, as conducted by Hassan et al. (2025), all modified starches were used and selected in sizes native starch (NS, 7840 nm), ultrasonicated starch (US, 231 nm), enzymatically modified starch (ES, 230.4 nm), and enzymatically sonicated starch (E‐US, 25 nm). Further, all reagents and chemicals utilized were of analytical grade and procured from Sinopharm Co. Ltd. (Shanghai, China).

### 2.2 Method of Analysis

#### 2.2.1 Characterization of Starch and Starch Nanoparticle

##### 2.2.1.1 Size Distribution

NS and E‐US samples' size distributions were assessed using dynamic light scattering (DLS) at 633 nm. Prior to analysis, the samples were diluted in deionized water at a concentration of 2 mg per 10 mL. The measurements were conducted at 30°C on samples without filtration or dust removal (Zhou et al. 2014).

#### 2.2.2 Preparation of Films

In preparation of NSCF by biocomposite film‐casting technique, a sample of 5 g of navy bean starch was added to 2.5 mL of glycerin. Then, complete the volume up to 100 mL of distilled water. Further, preparation of E‐USCF involved the mixing of 5 g of native starch with nanostarch at varying concentrations (0.5%, 1%, 2%, 5%, and 10%); 2.5% (w/w) glycerin was added to distilled water. Then, the volume of deionized water was completed up to 100 mL. After that, the solutions were gelatinized before being poured into Petri dishes. After drying at 50°C for 10 h (Dularia et al. 2019), the films were carefully peeled off (Figure S1).

#### 2.2.3 Mechanical Properties of Films

##### 2.2.3.1 Film Thickness

As reported by Fan et al. (2016), the thickness of biocomposite films in the size of 2.2 cm was measured with a caliper (Shanghai Shenhan Measurement Tools Co. Ltd., China) at various measurement points.

##### 2.2.3.2 Breaking Strength

The breaking strength of biocomposite films was measured with a texture analyzer, model TA.XY Plus (Stable Microsystems, Surrey, England). Then, each carrier was set with three‐centimeter film samples. After that, a ball probe (SMS P/0.25S, 1/4‐in. diameter) was contacted to a load cell. And the samples were settled between them. As reported by Roy et al. (2020), the breaking strength was measured by the maximum force on the curve.

##### 2.2.3.3 Tensile Strength

The strain–stress properties of the biocomposite films were measured by a testing machine (H25KS, Hounsfield, England) at a crosshead speed of 10 mm/min. Samples were taken, and a rectangular strip of 15 mm × 100 mm was made with a sharp hand press. The samples were optimized at 50% relative humidity (RH) and 23°C for 1 week before testing. The tensile test was done according to ASTM standard D882‐97 (Raj et al. 2021). And energy density was measured by the following equation:

$$U=\frac{1}{2}\mathrm{\sigma ɛ}$$

whereas *U* was the density, J/m^3^ or N.m/m^3^; σ was the stress *N*/m^2^; ɛ was the strain m/m.

#### 2.2.4 Physiochemical Properties of Films

##### 2.2.4.1 Water Vapor Transmission Rate (WVTR)

A sample of biocomposite films, 5 × 5 cm, was placed into a container filled with calcium chloride, and the container was placed in a desiccator set at 37°C, containing potassium nitrate (Shaikh et al. 2021). The biocomposite films were weighed daily for 5 days. According to Shafiee and Tabari (2018), the water vapor permeability (WVTR) was measured by the following equation:

$$\text{WVTR}=\frac{\Delta W\times X}{A\times T}$$

whereas WVTR was the water vapor transmission rate; *x* was the average thickness of the film; *A* represents the permeation area, and *x*/*t* was measured by linear regression from the points of weight gain and time during a constant‐rate period.

$$\mathrm{WVP}=\frac{\text{WVTR}\times Y}{\mathrm{\Delta p}}$$

whereas WVP was the water vapor permeability; *Y* was the average thickness of the film and Δ*p* is the difference in water vapor pressure on the two sides of the film.

##### 2.2.4.2 Determination of pH

As reported by Raj et al. (2023), the pH value of biocomposite films was measured with a digital pH meter (pHs‐3BW) and adjusted with standard buffer solutions of pH values 4 and 7. Thus, according to Soydal et al. (2023), a sample of biocomposite films, a 25 × 25 mm piece cut from samples, was set in 20 mL of 0.9% sodium chloride for 3 days. Then, each sample was weighed every 24 h, and the average was calculated.

##### 2.2.4.3 Moisture Content

As reported by Oluwasina et al. (2021), a 2 cm sample of biocomposite film was dried in a hot oven at 100°C for 3 h. The biocomposite films were reweighted to obtain a constant and final weight by the following equation:

$$M=\left(W0–W\right)/W0\times 100\%$$

whereas *M* was the moisture content, 100%; *W*0 was the sample's initial weight, *g*, and *W* was the sample's final weight, *g*.

##### 2.2.4.4 Determination of Color

The color values for biocomposite films were assessed using a Chroma meter CR 200b (Minolta Camera Co. Ltd., Tianjin, China). This instrument measured three color parameters: L* (lightness), a* (redness), and b* (yellowness). The total color difference (Δ*E*) between the samples was calculated using the following equation (Olawuyi and Lee 2022):

$$\Delta E=\sqrt{{\left(L_{o}-L^{*}\right)}^{2}\;+{\left(B_{o}-B^{*}\right)}^{2}+{\left(A_{o}-A^{*}\right)}^{2}}$$

whereas Δ*E* indicated a significant deviation in color; o indicated color reading of the control sample; L* was the lightness spectrum; a* was the green to red spectrum; b* The blue to yellow spectrum.

##### 2.2.4.5 Determination of Transparency

A sample cut of 10 × 30 mm from biocomposite films in a rectangular shape was measured with a UV spectrophotometer (Lambda25, PerkinElmer Instrument Co. Ltd., Tianjin, China). Samples' transparency was determined by an absorbance of 600 nm. Each measurement was conducted in duplicate (Vejdan et al. 2016). The transparency of the films was calculated by the following equation:

$$\text{Transparency}=\frac{A600}{T}\times 100\%$$

whereas *A*600 was the absorbance at 600 nm; *T* is the average film thickness (mm).

##### 2.2.4.6 Determination of Swelling Index

A biocomposite film's cut in the size of 2.2 cm was dried for 3 h at 100°C and weighed (A). After drying, the sample was submerged in 50 mL of deionized water for 5 min at room temperature. Then, the sample was filtered to remove excess water by Whatman filter paper. Finally, the swollen sample was reweighed (B). As described by Avérous et al. (2001), the swelling property of the sample was calculated using the following equation:

$$S_{W}=\frac{\left(A-B\right)}{A}\times 100\%$$

whereas *SW* was the Swelling power, 100%; *A* was the weight of absorbed sample, g; *B* was the weight of dried sample, g.

##### 2.2.4.7 Determination Water Solubility of the Film

After the biocomposite films were dried in a hot air oven at 105°C for 3 h, their original weight (2 cm × 2 cm) was determined. After that, the dry films were agitated at 100 r/m for 24 h at room temperature while submerged in 15 mL of distilled water. After drying the water‐soaked films once more for 3 h, the final weight of the films (2 × 2 cm) was determined. Once more, the dried samples were weighed. The following equation was used to calculate the samples' solubility (Wang et al. 2017).

$$\text{Water solubility}=\frac{\text{Intial weight of the sample}-\text{final weight of the sample}}{\text{Intial weight of the sample}\;}\times 100$$

##### 2.2.4.8 Biodegradability Tests

A sample of biocomposite film, a 5 × 5 cm, was buried at a depth of 3 cm from the soil surface. Then, the samples were weighed every 2, 4, 6, and 8 days by an analytical balance. After each weighing, the samples were dried at 40°C for 15 min and thoroughly cleaned from any residual dirt. After awards, they were placed in a desiccator for 1 day to stabilize their weight (Othman et al. 2023). The decomposition rate (g/d) and reduced weight percentage were measured by the following equation:

$$\mathrm{Ws}=\frac{W0-W}{W0}\times 100\%$$

whereas *W*_*S*_ indicated Weight loss, 100%; *W*_0_ was the film sample's initial weight, g; *W* was the film sample's weight following each test periodically, g.

$$D_{G}=\frac{\mathrm{Ws}}{T}$$

whereas *DG* was the rate of degradation, g/day; *WS* indicated weight loss of film sample, g; *T* was the time for samples' weight loss per day.

#### 2.2.5 Thermal Properties

A sample of biocomposite film, 3 mg, was placed in a small aluminum oxide plate. Then, it was measured with a thermal analyzer (STA 409PC, Netzsch, Weimar, China). As reported by Raj et al. (2023), a temperature ranged from 30°C to 500°C at an average rate of 10°C/min and a nitrogen flow rate of 30 mL/min. Nitrogen was used as the purge gas, and the heating rate was set at 10°C/min.

### 2.3 Statistical Analysis

The obtained data were analyzed by analysis of variance (ANOVA) using XLSTAT software version 2014, 5.03 (Addinsoft, New York, NY, USA) in three repeats. It was expressed as the mean ± standard error of the mean. The significance of variance between means of samples was calculated at *p* value ≤ 0.05, which was considered significant.

## 3 Results and Discussion

### 3.1 Dynamic Light Scattering (DLS)

**FIGURE 1:**
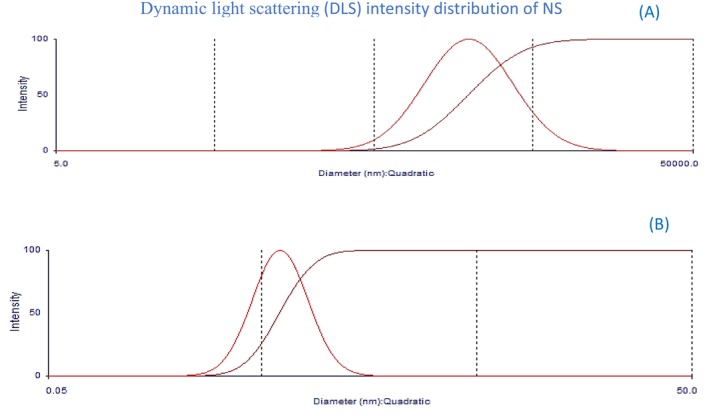
Dynamic light scattering for hydrodynamic diameter of native (A) and nano (B) navy bean starch.

The size distribution of NS and E‐US as determined by dynamic light scattering (DLS) was illustrated in Figure 1. Figure 1A showed that the NS has a single peak with an intensity of 100% and a size of approximately 7480 nm. In contrast, Figure 1B depicts the size distribution of E‐US, which had a size of 25 nm at 100% intensity, significantly smaller than the NS. The selection of a size of 25 nm was conducted according to the study of Hassan et al. (2025), who reported that obtaining modified navy bean starch in nanosized was through enzymatically debranching in 15 min and a sonication process at 450 watts. Therefore, when the physical composition of navy bean starch was altered by ultrasonic cavitation, the starch molecular chains' capacity to split improved, the viscosity of the starch paste decreased, and pullulanase, an enzyme that debranched starch, was able to alter the chemical structure of the E‐US sample. Therefore, the E‐US sample's size at 25 nm was chosen based on a reasonable amylose percentage and a uniform dispersion of nanoparticles based on the sample's poly dispersion index (PDI). Additionally, the enzymatic debranching process took 15 min. According to Raghunathan et al. (2025), Starch retrogradation was increased by prolonged debranching times, which contributed to the formation of larger starch granules. Additionally, as described by Hassan et al. (2025), amylose molecules most likely formed double helices through intermolecular hydrogen bonding during the retrogradation process. Besides that, amylopectin has a dendritic structure and significant spatial obstacles in solution, which made it difficult to orient (Tian and Sun 2020).

### 3.2 Thickness, Water Vapor Transition Rate, and Breaking Strength of Biocomposite Films

As demonstrated in Table 1, samples of 2%, 5%, and 10% E‐USCF showed significant thickness increased (*p* < 0.05) compared to NSCF. Further, there is no significant difference in thickness for 0.5% and 1% E‐USCF samples. According to Sharma et al. (2021), increased concentrations of nanostarch led to thick kidney bean starch biocomposite films. As reported by Fan et al. (2016)., the solid content of the film‐forming dispersion led to uniform thickness, which was essential for the consistent and burst strength of biocomposite films.

**TABLE 1:** The thickness, water vapor transition permeability, and breaking strength of starch biocomposite films.

Samples	Thickness (mm)	WVTR (g/m^2^/s)	Breaking strength (N/m^2^)	WVP (g m m^−2^ s^−1^ Pa^−1^)
Native starch film (NSCF)	0.18 ± 0.06^d^	6.1 × 10^−4^ ± 0.5^a^	1578.33 ± 52.60^d^	8.66 × 10^−8^ ± 0.42^a^
E‐USCF (0.5%)	0.15 ± 0.01^d^	4.8 × 10^−4^ ± 0.6^b^	1606.77 ± 59.79^d^	5.72 × 10^−8^ ± 0.42^b^
E‐USCF (1%)	0.16 ± 0.03^d^	3.9 × 10^−4^ ± 0.4^c^	2000 ± 50.03^c^	5.25 × 10^−8^ ± 0.43^b^
E‐USCF (2%)	0.22 ± 0.02^c^	3.8 × 10^−4^ ± 0.3^c^	2176 ± 56.01^b^	4.86 × 10^−8^ ± 0.44^c^
E‐USCF (5%)	0.31 ± 0.05^b^	3.1 × 10^−4^ ± 0.5^d^	2200 ± 40.41^b^	4.65 × 10^−8^ ± 0.41^c^
E‐USCF (10%)	0.40 ± 0.02^a^	2.8 × 10^−4^ ± 0.4^d^	2600 ± 50^a^	4.44 × 10^−8^ ± 0.40^c^

*Note:* Lowercase letters indicated significant differences (*p* < 0.05) in the experimental results (means and SD for *n* = 3).

As shown in Table 1, E‐USCF's WVTR and WVP samples decreased significantly (*p* < 0.05) compared to NSCF samples. Thus, the obtained results were consistent with the findings of Sharma et al. (2021), who showed that an elevation in nanostarch amount declined the WVTR of kidney bean biocomposite films. Additionally, Chavan et al. (2022), stated that a WVP average of native starch‐based film was high in comparison to the other samples as a result of the reinforcement of cross‐linked starch nanoparticles.

In addition, as presented in Table 1, NSCF was significantly (*p* < 0.05) the lowest in breaking strength, but E‐USCF (10%) recorded the highest one rather than other samples, and the increase in breaking strength through the inclusion of nanoparticles improved stiffness and rigidity of bio composite films. And this could be as a result of amylose chains breaking down during enzymatic hydrolysis of navy bean starch (Hassan et al. 2025). Additionally, as observed by Qiu et al. (2016), reinforcement of debranched waxy maize starch nanoparticles in biocomposite films increased breaking strength by 85.9%. In addition, as reported by (Marta et al. 2022; Gamage et al. 2022), starch nanoparticles (SNPs) as a reinforcing filler improved the breaking strength of bio composites. Since they were rich in hydroxyl groups, which served as crosslinking sites produced strong intermolecular bonds with the polymer matrix because of a large surface area to volume ratio, which reduced chain mobility and improved breaking strength.

### 3.3 Moisture Content, pH, Film Color and Transparency

As presented in Table 2, the amount of moisture for biocomposite films highlighted a significant difference (*p* < 0.05) between NSCF and E‐USCF samples. Because E‐USCF samples of 5% and 10% had the lowest moisture content, while 0.5%, 1%, and 2% E‐USCF samples did not have significant differences in values compared to the native one. As reported by Castillo et al. (2017), the high moisture content of traditional biocomposite film was linked with its hygroscopic and hydrophilic properties, while usage of nanosized fillers reduced the moisture content in the biocomposite matrix. Furthermore, according to Sharma et al. (2021), they observed similar results with kidney bean nanostarch films. Since moisture content affected the mechanical characteristics of starch‐based materials, adding fillers improved film structure and reduced moisture exposure. In addition, Oluwasina et al. (2021), stressed the need for food packaging materials with low moisture content to prevent contamination, and it reflected an increase in film thickness.

**TABLE 2:** Biocomposite film's pH, moisture content (%), color and transparency.

Samples	pH	Moisture content (%)	Color				Transparency (%)
			L*	a*	b*	∆*E*	
Native starch film (NSCF)	5.52 ± 0.36^a^	10.57 ± 0.49^a^	2.23 ± 0.21^b^	1.2 ± 0.1^a^	4.90 ± 0.1^a^	5.33 ± 0.30^a^	29.10 ± 2.06^a^
E‐USCF (0.5%)	5.84 ± 0.60^a^	8.60 ± 0.75^b^	3.80 ± 1.39^a^	1.30 ± 0.96^a^	5.40 ± 2.23^a^	6.90 ± 0.45^a^	30.36 ± 2.06^a^
E‐USCF (1%)	5.87 ± 0.38^a^	8.09 ± 0.16^b^	44.00 ± 0.99^a^	1.30 ± 0.2^a^	5.70 ± 0.82^a^	7.05 ± 1.03^a^	29.70 ± 2.06^a^
E‐USCF (2%)	6.04 ± 0.45^a^	8.05 ± 0.12^b^	4.10 ± 1.65^a^	1.40 ± 0.37^a^	5.80 ± 1.16^a^	7.08 ± 1.27^a^	24.13 ± 2.20^b^
E‐USCF (5%)	6.18 ± 0.57^a^	7.43 ± 0.16^c^	4.30 ± 0.71^a^	1.50 ± 0.36^a^	5.90 ± 0.23^a^	7.54 ± 0.53^a^	13.00 ± 2.00^c^
E‐USCF (10%)	6.22 ± 0.91^a^	6.82 ± 0.13^d^	4.60 ± 1.4^a^	1.90 ± 0.42^a^	5.90 ± 0.85^a^	7.61 ± 1.63^a^	8.47 ± 0.15^d^

*Note:* Lowercase symbols denoted significant differences (*p* < 0.05) at the experimental variations (means and SD to *n* = 3).

As shown in Table 2, NSCF was in a slightly acidic state. In contrast, the pH value of E‐USCF samples increased gradually by raising nanostarch concentration. As reported by Sakkara et al. (2020), the packaging industry of biocomposite films was preferred to have a pH close to 7. As suggested by Raj et al. (2023), biocomposite films produced in an alkaline medium were better suited for packaging.

As presented in Table 2, there were no significant variations between the color of NSCF and E‐USCF samples. As reported by AlAseebee et al. (2021), starch nanoparticles did not affect the biocomposite film's colors and compactness. As shown in Table 2, the transparency value of NSCF was reduced significantly (*p* < 0.05) with an increase in the amount of nanostarch concentration. Thus, the results of transpiration were linked with the increase in thickness and pH values of E‐USCF samples. Additionally, according to Zhao et al. (2020), the increased film thickness and pH values in high nanostarch content were linked with greater opacity. In a consequence, as described by Kumari et al. (2021), emphasized that reduced transparency could prevent the oxidation of fats, oils, and pigments and minimize vitamin loss.

### 3.4 Swelling Index and Water Solubility of Biocomposite Films

**FIGURE 2:**
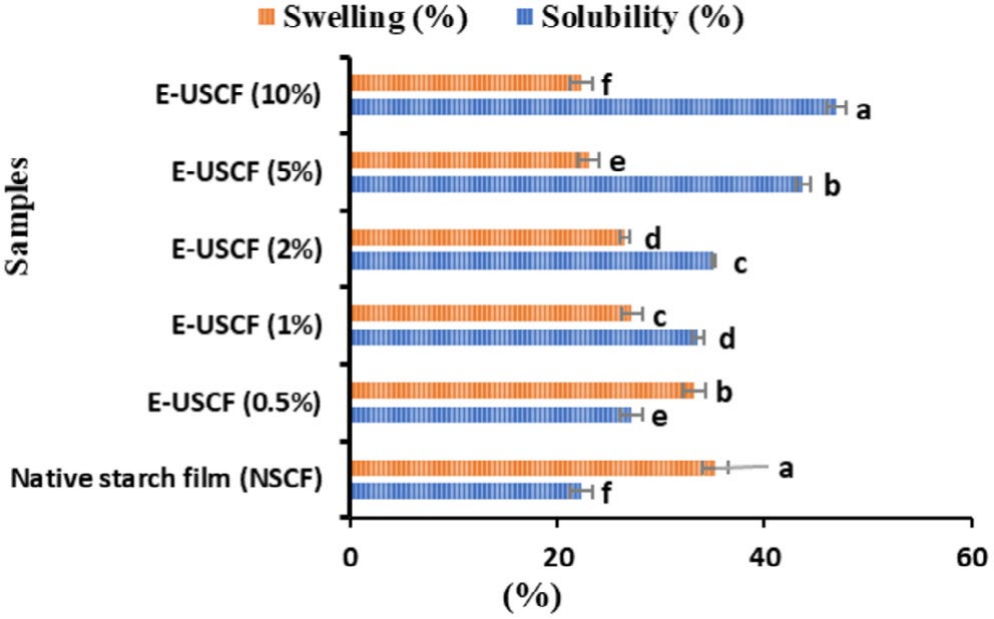
The swelling index and water solubility for NSCF and E‐USCF are evaluated at treatment levels of 0.5%, 1%, 2%, 5%, and 10%. Whereas NS referred to native starch, US represented ultrasonic modification of navy bean starch, ES indicated enzyme debranching of navy bean starch, and E‐US stands for ultrasound‐assisted enzyme debranching used to produce starch nanoparticles. Lowercase symbols denoted significant differences (*p* < 0.05) at the experimental variations (means and SD to *n* = 3).

As illustrated in Figure 2, the swelling property of NSCF was 35.25% ± 1.25. As reported by Mathew et al. (2025), the inclusion of hydrophilic groups in biopolymers influenced the swelling property of biocomposite films through the interaction between water molecules and starch particles. Although biocomposite films with increased nanostarch reinforcement concentrations exhibited significantly lower swelling indices (*p* < 0.05), they ranged from 33.26% ± 1.09 to 22.27% ± 1.10. According to Versino et al. (2016), biocomposite films should have low swelling capacity to minimize water penetration and prevent forming bonds of hydrogen between molecules of starch.

As shown in Figure 2, the initial solubility for NSCF was 22.27% ± 1.10. In contrast, solubility of E‐USCF samples increased significantly (*p* < 0.05) in a range between 27.23% ± 1.08 and 46.98% ± 0.97. Sharma et al. (2021) also composed biocomposite films using kidney bean starch in concentrations of nanostarch ranging from 0.5% to 10% with an elevation in solubility and decline in WVTR compared to the native one; this might be due to the nanometric size of the starch, which raised the surface volume ratio and interacted with polymer mobility (Fan et al. 2016). Besides, as described by Ye et al. (2019), enzymolysis broke chains (glycosidic bonds broke), increased molecular polarity, and declined hydrogen bonds between chains, which might explain the main reason for high solubility. On the other hand, this result was compiled with the decrease in values of moisture and WVTR. As described by Emamifar and Bavaisi (2020), the nanometric size led to a decrease in the WVTR of biocomposite films owing to the influenced surface area. Because of the addition of nanoparticles within the starch films, enhancing moisture resistance leads to a hard pathway for water passing within the biocomposite films. As demonstrated by Monteiro et al. (2018), the moisture barrier was composed of the completion of nanocrystals in the biocomposite film, which declined the diffusion of water particles throughout the film. Beyond that, as exhibited by Dey and Sit (2017), when various techniques were combined, the solubility of starch rose significantly and starch particles degraded. On the other hand, native kidney bean film showed the lowest solubility since a high amount of amylopectin lowered film solubility in water, resulting in the accumulation of starch microgranules (Thakur et al. 2019).

### 3.5 Biodegradability of Biocomposite Films

**FIGURE 3:**
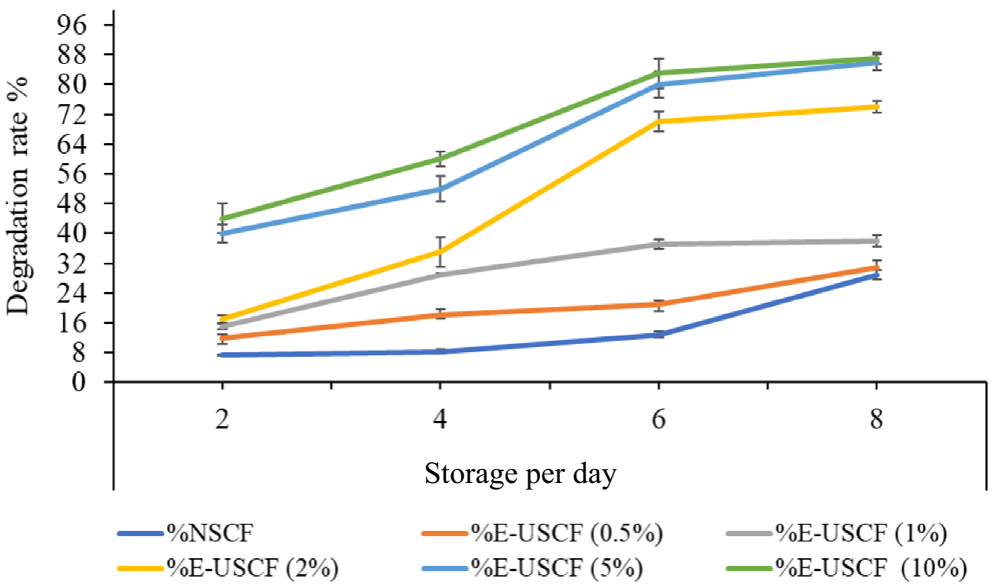
The biodegradation rate for NSCF and E‐USCF is assessed at treatment levels of 0.5%, 1%, 2%, 5%, and 10%. Whereas NSCF and E‐USCF showed the native starch biocomposite film and ultrasonicated enzymatically modifying starch biocomposite film, respectively. Lowercase symbols denoted significant differences (*p* < 0.05) at the experimental variations (means and SD to *n* = 3).

As shown in Figure 3, the degradation rate value for the NSCF sample within 8 days ranged from 7.2% ± 0.15 to 29% ± 1.26. On the other hand, for E‐USCF samples, the degradation rates for 0.5% E‐USCF samples fell between 2% ± 0.76 and 31% ± 1.76; 1% E‐USCF samples ranged from 15% ± 0.76 to 38% ± 1.61; 2% E‐USCF samples ranged between 17% ± 1.00 and 74% ± 1.52. 5% E‐USCF samples ranged between 40% ± 2.50 and 86% ± 2.08, and 10% E‐USCF samples fell between 44% ± 4.00 and 87% ± 1.52. Which means biodegradation rates of biocomposite films increased significantly (*p* < 0.05) within 8 days with high concentrations of nanostarch. As reported by Marta et al. (2022), an elevation in biodegradability rates was caused by the usage of starch nanoparticles because of a decrease in the packing polymer's molecular mass, which sped up the process of degradation and frequently caused greater weight loss in soil burial studies.

### 3.6 Thermal Properties

#### 3.6.1 Thermogravimetric Analyses (TGA)

**FIGURE 4:**
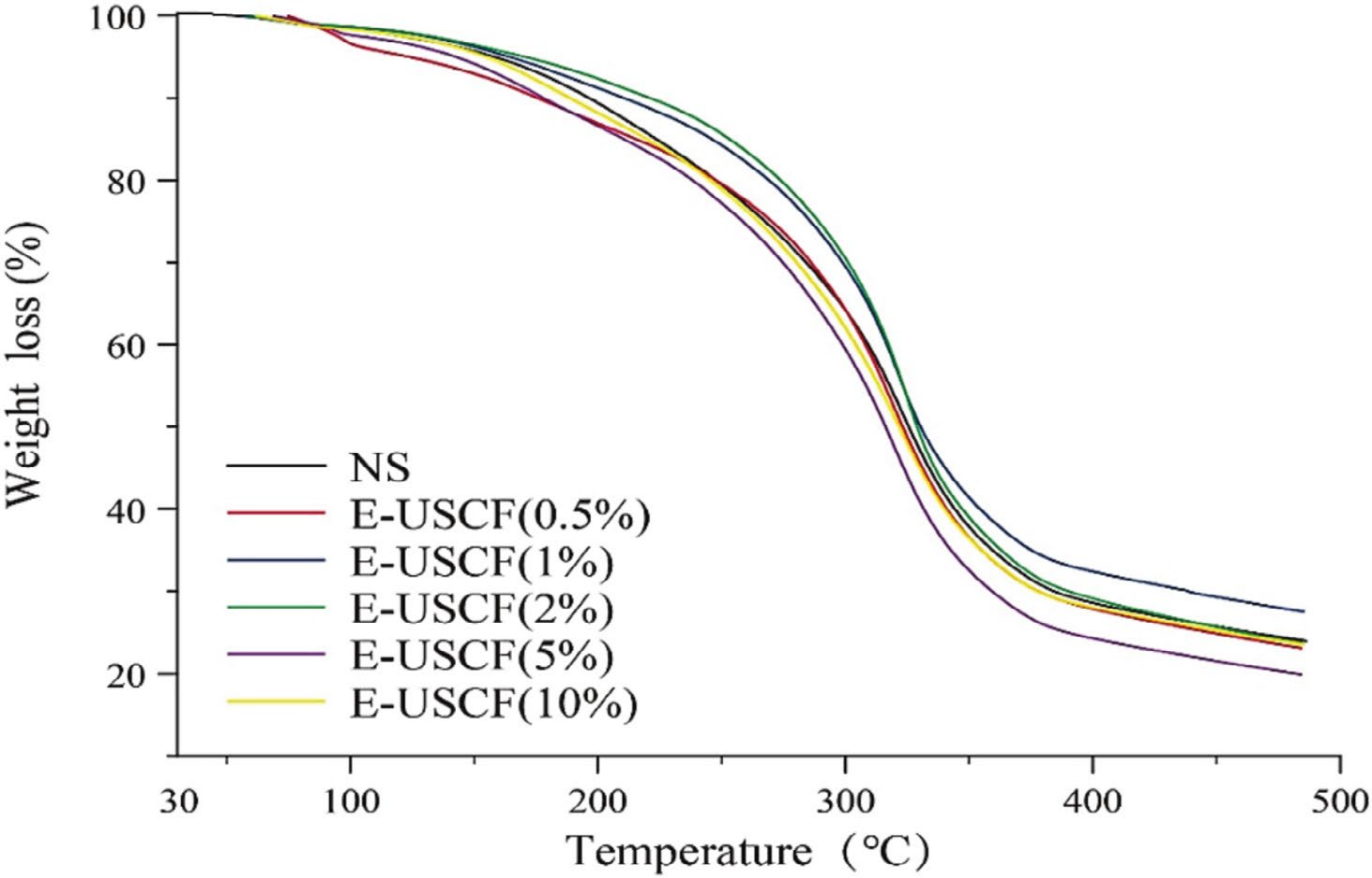
Thermogravimetry of NSCF and E‐USCF at 0.5%, 1%, 2%, 5%, and 10%. Whereas NSCF and E‐USCF showed the native starch biocomposite film and ultrasonicated enzymatically modifying starch biocomposite film, respectively.

As presented in Table 3, biocomposite films exhibited distinct phases of heat degradation. The first phase, ranging from 30°C to 101.51°C, corresponded to the evaporation of small molecules, resulting in a decrease in moisture. The second phase was linked to starch degradation due to depolymerization in starch components of the biocomposite films and occurred at temperatures ranging between 101.51°C and 292.46°C. Those findings were aligned with the results of (Shapi'i et al. 2022). As presented in Table 3 and Figure 4, biocomposite films exhibited distinct phases of heat degradation. The first phase, ranging from 30°C to 101.51°C, corresponded to the evaporation of small molecules, resulting in a decrease in moisture. The second phase was linked to starch degradation due to depolymerization in starch components of the biocomposite films and occurred at temperatures ranging between 101.51°C and 292.46°C. Those findings were aligned with the results of (Shapi'i et al. 2022).

**TABLE 3:** TGA parameter values for navy bean starch biocomposite films, corresponding onset (T_o_), derivative of weight loss (Td) and end set (Tc) temperatures.

Film samples	T_o_ (°C)	T_d_ (°C)	End set (°C)	Ash residual (%)
NSCF	63.37	250.90	352.41	23.59
E‐USCF (0.5%)	78.28	270.61	353.56	23.61
E‐USCF (1%)	82.95	292.46	355.83	23.59
E‐USCF (2%)	101.51	281.05	359.33	23.19
E‐USCF (5%)	98.08	262.48	357.88	21.00
E‐USCF (10%)	95.40	254.39	352.47	19.88

A secondary degradation phase ranged from 250.90°C to 359.33°C. As reported by Kim et al. (2018), degradation of azuki bean starch and starch films containing starch nanoparticles was due to structural changes in the film network. The third phase of degradation occurred above 350°C. Furthermore, the ash residual's value for the NSCF sample was 23.95%. But the value of ash residual was decreased to 19.88% by an elevation in the concentration of the E‐US sample up to 10% because of the decrease in the structure of the biocomposite films. According to Liu et al. (2016), the incorporation of starch nanoparticles into starch films decreased the value of ash residual from 10% to 23%. Additionally, these results were in line with the increase in biodegradability of the E‐USCF samples.

### 3.7 Tensile Strength

**FIGURE 5:**
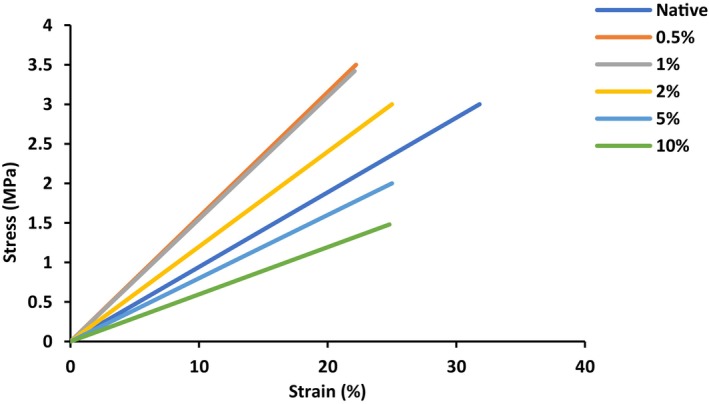
Stress–strain curve of NSCF and E‐USCF at 0.5%, 1%, 2%, 5%, and 10%. Whereas NSCF and E‐USCF showed the native starch biocomposite film and ultrasonicated enzymatically modified starch biocomposite film, respectively.

As shown in Figure 5 and Table 4, biocomposite samples bearing demonstrated a decrease in the stress by a reduction in the strain rate compared to the NSCF sample. Therefore, nanostarch was a low‐density polymeric reinforcement, and when it was reinforced into biocomposite films, it could impart good mechanical behavior. According to Callister and Rethwisch (2022), low‐density polyethylene (LDPE) was characterized by a relationship between relatively low stress and strain because of its flexible polysaccharide structure and limited load‐bearing capability. In addition, this was associated with the incorporation of debranched and sonicated starch nanoparticles. Also, as described by Sun et al. (2025), the existence of an active functional hydroxyl group on its surface interacted with the starch to compose a rigid network.

**TABLE 4:** The starch biocomposite film's Stress (Mpa) and Strain (%).

Samples	Stress (Mpa)	Strain %
NSCF	3.00^b^	31.81^a^
E‐USCF 0.5%	3.50^a^	22.20^b^
E‐USCF 1%	3.42^a^	22.10^b^
E‐USCF 2%	3.00^b^	25.00^c^
E‐USCF 5%	2.00^c^	25.00^c^
E‐USCF 10%	1.48^d^	24.80^d^

*Note:* Lowercase symbols denoted significant differences (*p* < 0.05) at the experimental variations (means and SD to *n* = 3).

**FIGURE 6:**
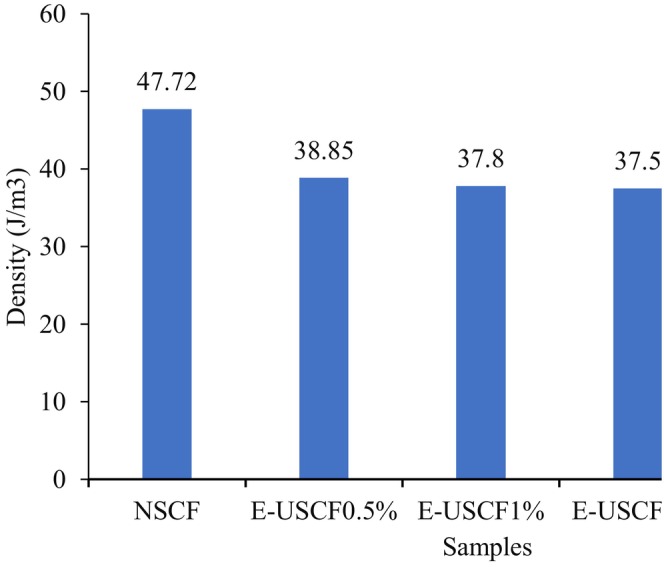
Density of NSCF and E‐USCF at 0.5%, 1%, 2%, 5%, and 10%. Whereas NSCF and E‐USCF showed the native starch biocomposite film and ultrasonicated enzymatically modifying starch biocomposite film, respectively.

As shown in Figure 6, with the increase of starch nanoparticle reinforcement in the biocomposite matrix, there was a decrease in the density of the biocomposite matrix. As supported by Musa and Hameed (2020), starch had amorphous properties, and the reduction in the value of tensile stress of the biocomposite matrix was caused by a decrease in the density of hydrogen bonds in the polymer matrix.

## 4 Conclusion

The study indicated that E‐USCF had the potential to produce environmentally friendly and biodegradable starch biocomposite films suitable for industrial applications. So, according to the E‐US sample's characteristics, the E‐US particles were applied for the production of biocomposite films (E‐USCF) in different concentrations. To sum up, a 10% E‐USCF sample with high solubility offered lower water resistance compared with the NSCF sample, which will be beneficial for applications in low‐melting‐temperature biodegradable packaging. Therefore, a 10% E‐USCF sample had the lowest stress, strain, and density of hydrogen bonds compared to the native one. This was due to a decrease in the molecular weight of the utilized modified starch nanoparticles. So, further modifications and optimizations are required to enhance the films' functionality for more types of biodegradable packaging.

## 5 Recommendations and Future Prospects

Research was conducted on the utilization of nano starch as a nanofiller in the production of biocomposite films, aimed at enhancing performance, lowering the number of raw materials used, and reducing environmental effects. Additionally, it was essential to raise consumer knowledge and acceptance of starch‐based biopolymers as eco‐friendly substitutions for traditional plastics to be used as food packaging. In this study, production of biocomposite films was determined under laboratory conditions only as a small‐scale industry, but further investigations are required to determine its potential for industrial output. So, recognizing companies and consumers about the importance of sustainability, recyclable nature, and biodegradability of packaging materials as a real study will promote wider use and increase demand.

## Author Contributions

**Rawaa Houri Tlay:** data curation, software, methodology, conceptualization, writing – review and editing, writing – original draft, supervision. **Ammar Badran Altemimi:** conceptualization, data curation, writing – review and editing, software. **Bing Lu:** data curation, software, conceptualization, writing – review and editing. **Tarek Gamal Abedelmaksoud:** data curation, supervision, software, methodology, conceptualization, writing – original draft, writing – review and editing. **Aijun Hu:** conceptualization, writing – original draft, writing – review and editing, software, data curation, supervision, methodology. **Nora Ali Hassan:** conceptualization, methodology, writing – original draft, writing – review and editing, data curation. **Qian Zhang:** conceptualization, data curation, writing – review and editing, software. **Shiwei Liu:** data curation, software, writing – review and editing, conceptualization.

## Conflicts of Interest

The authors declare no conflicts of interest.

## Supporting information


**Figure S1:** Claim demonstration of starch‐based films with different nano‐starch concentrations: NSCF, E‐USCF0.5%, E‐USCF1%, E‐USCF2%, E‐USCF5%, and E‐USCF10%.

## Data Availability

Data will be made available on request.
