# Supplementary material for: Biocomposite Films Reinforced With Nano Navy Bean Starch: Application and Characterization
Source: Food Sci Nutr. 2026 Jul 7;14(7):e72067. doi: 10.1002/fsn3.72067 (PMC13338711; doi:10.1002/fsn3.72067)
Supplement: Supplementary file 1 — Figure S1: Claim demonstration of starch‐based films with different nano‐starch concentrations: NSCF, E‐USCF0.5%, E‐USCF1%, E‐USCF2%, E‐USCF5%, and E‐USCF10%. [file FSN3-14-e72067-s001.docx]

**Biocomposite Films Reinforced with Nano Navy Bean Starch: Application and Characterization**

**Nora Ali Hassan^a,b^, Aijun Hu^a,^ Ammar B. Altemimi ^c^, Rawaa H. Tlay ^d, *^, Qian Zhang^a^, Bing Lu^a^**, **Shiwei Liu^a^ and Tarek Gamal Abedelmaksoud^b, *^**

**Supplementary description**

**Figures:**

| 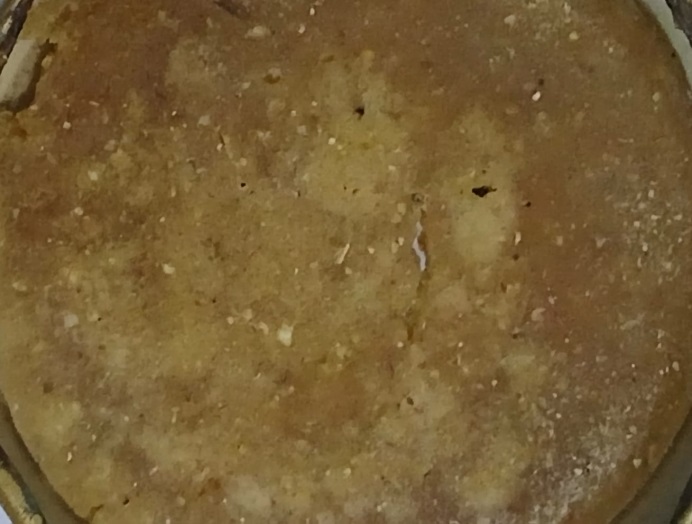  NSCF | 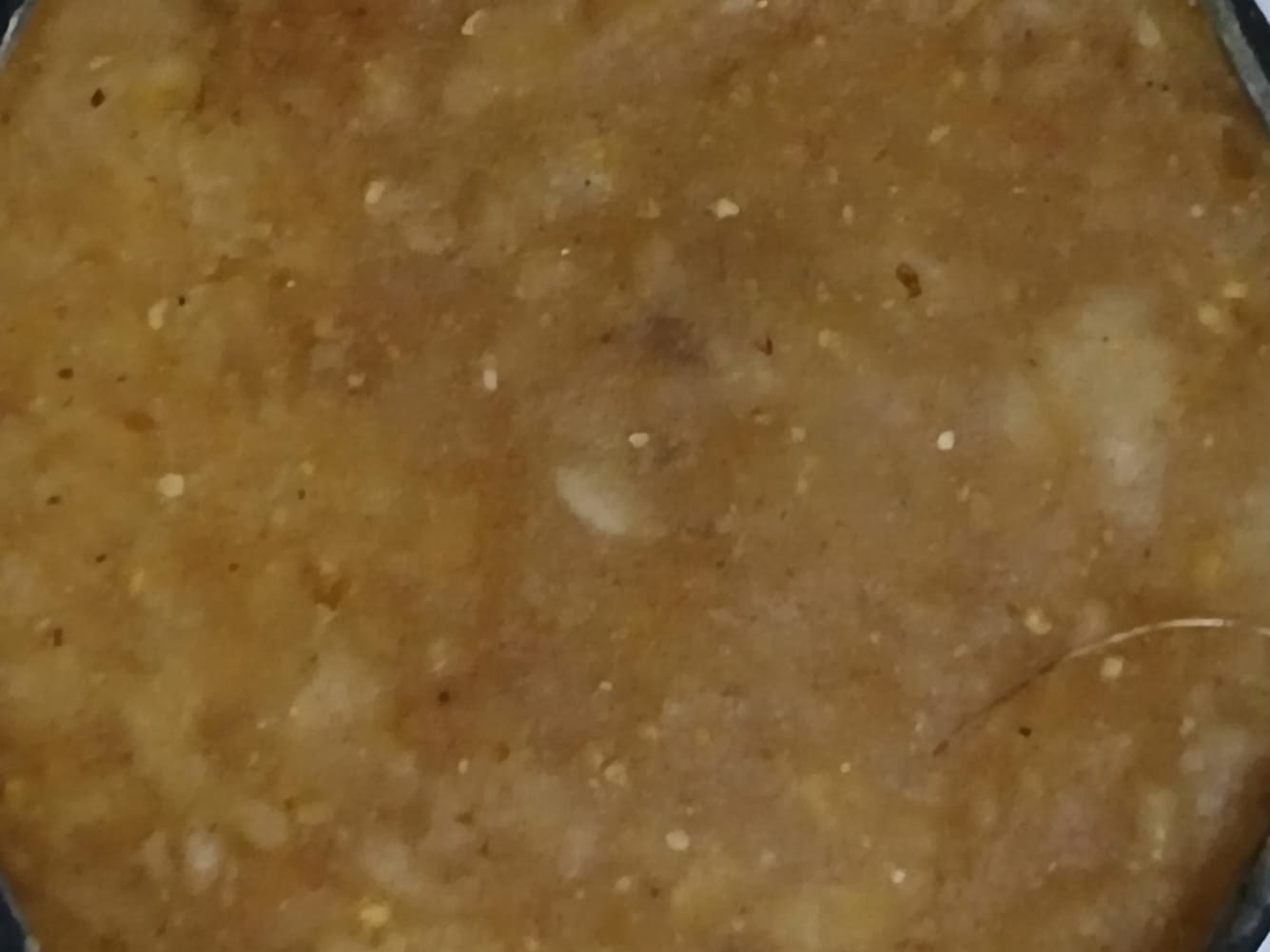  E-USCF0.5% |
| --- | --- |
| 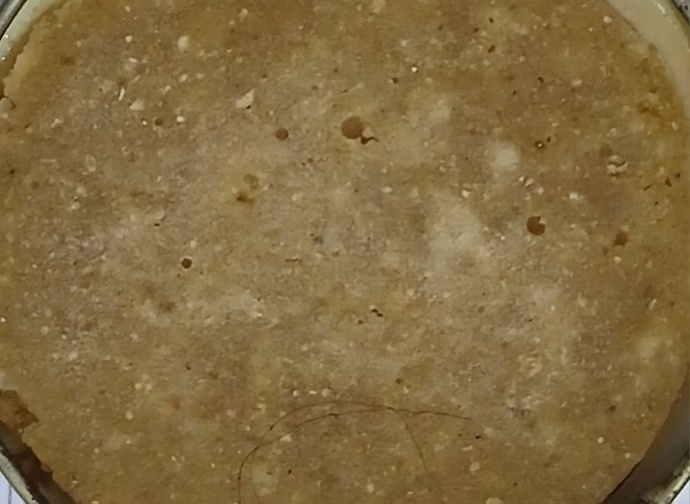  E-USCF 1% | 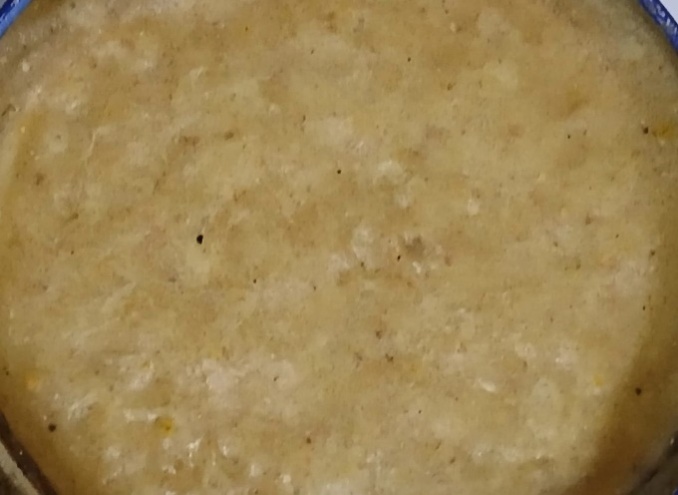  E-USCF 2% |
| 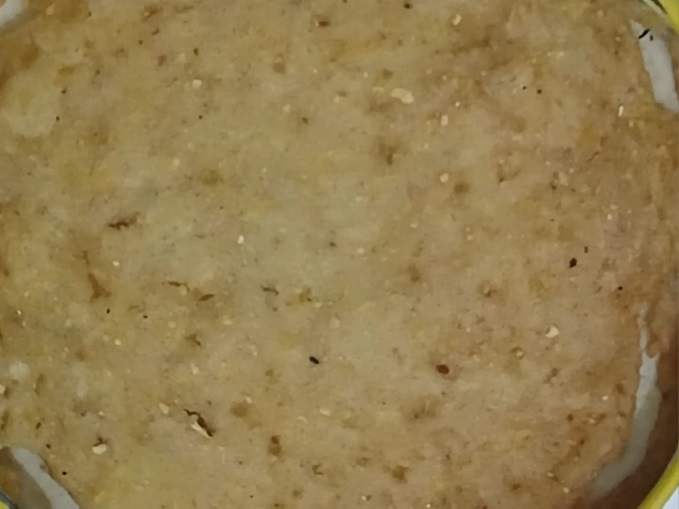  E-USCF 5% | 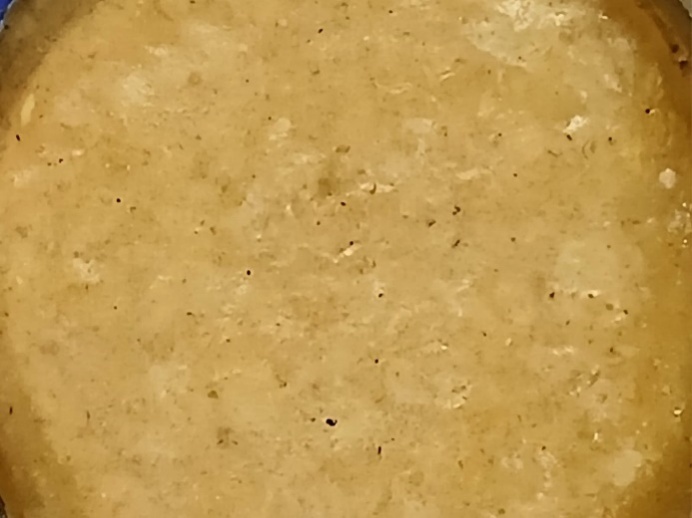  E-USCF 10% |

**Figure S1. Claim demonstration of starch-based films with different nano-starch concentrations: NSCF, E-USCF0.5%, E-USCF1%, E-USCF2%, E-USCF5%, and E-USCF10%.**
